# Supplementary material for: Trehalose 6-Phosphate/SnRK1 Signaling Participates in Harvesting-Stimulated Rubber Production in the Hevea Tree
Source: Plants (Basel). 2022 Oct 27;11(21):2879. doi: 10.3390/plants11212879 (PMC9655858; doi:10.3390/plants11212879)
Supplement: Supplementary file 1 [file plants-11-02879-s001.zip › Supplemental figures and talbes.pdf]

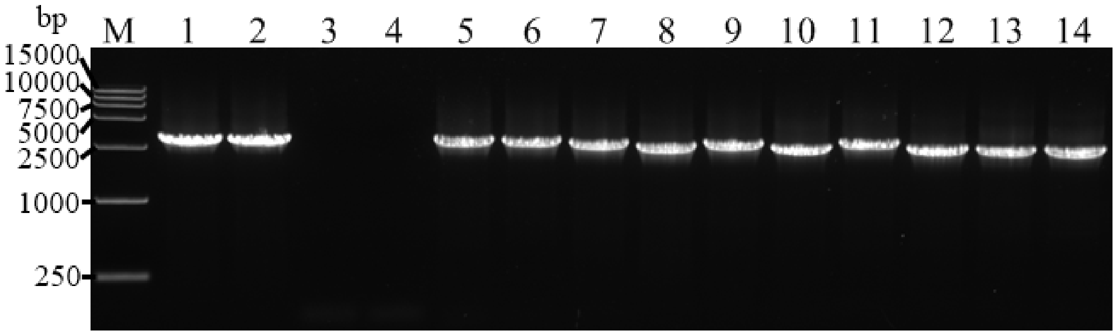

Fig. S1. Amplification of the full-length cDNAs of the *H. brasiliensis TPS* gene family.

Lanes: M, DL15000 DNA Marker; 1-14, the PCR products for the full-length cDNAs of *HbTPS1* to *14*, respectively. The RNA samples from different Hevea tissues were pooled in equal amounts, reverse transcribed and used as the template for PCR amplification. The full-length cDNAs of *HbTPS3* and *4* could not be amplified.

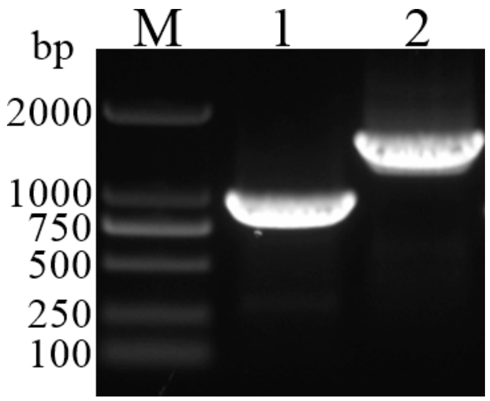

Fig. S2. Amplification of partial genomic sequences of *HbTPS3* and *4*.

Lanes: M, DL2000 DNA marker; 1-2, the PCR products for the partial genomic sequences of *HbTPS3* and *HbTPS4*, respectively.

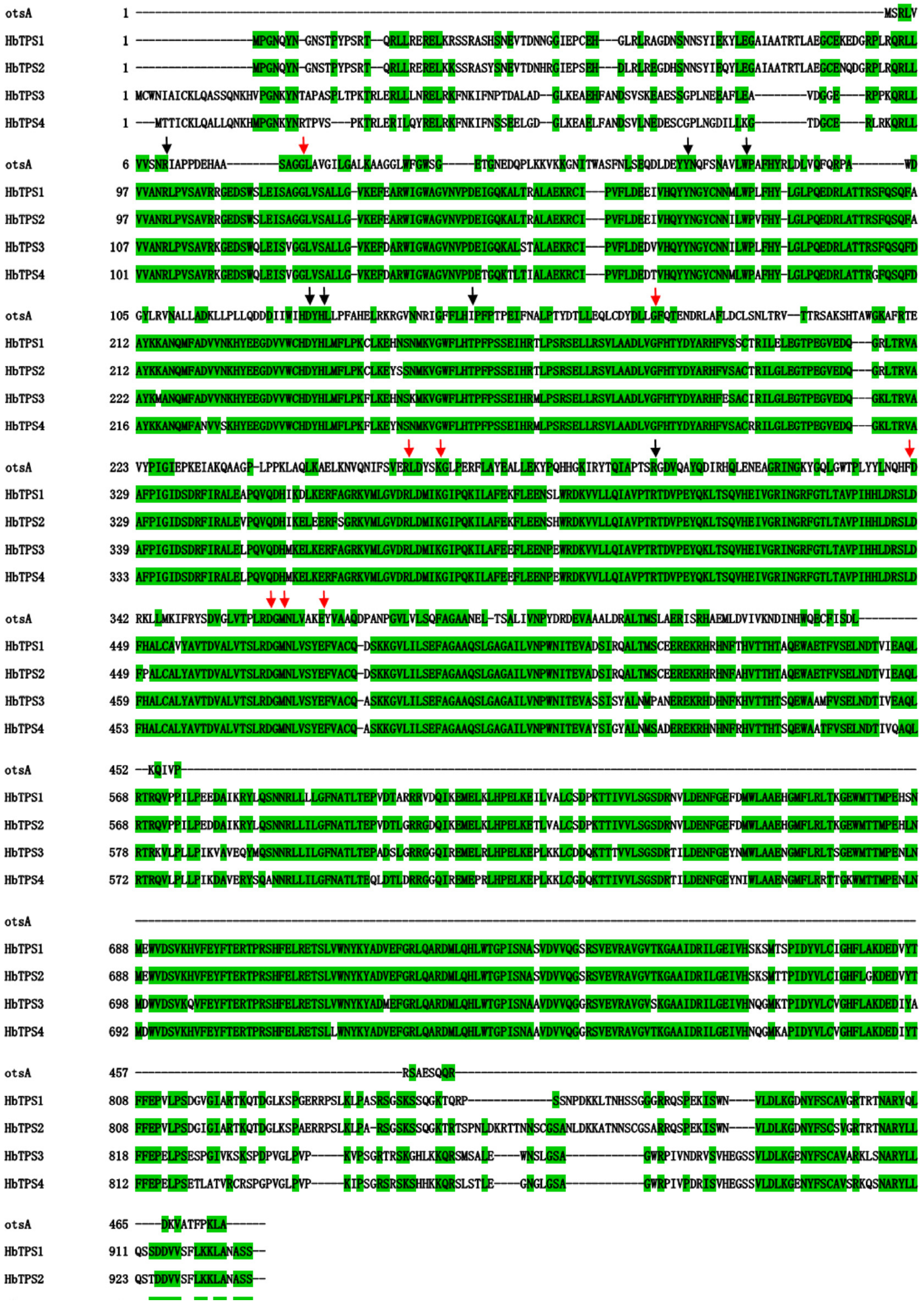

Fig. S3. Alignment of *E. coli* otsA with the *Hevea* class I TPSs (HbTPS1 to 4).

Black arrows indicate aa residues important for glucose-6-phosphate binding. Red arrows show residues important for UDPG binding.

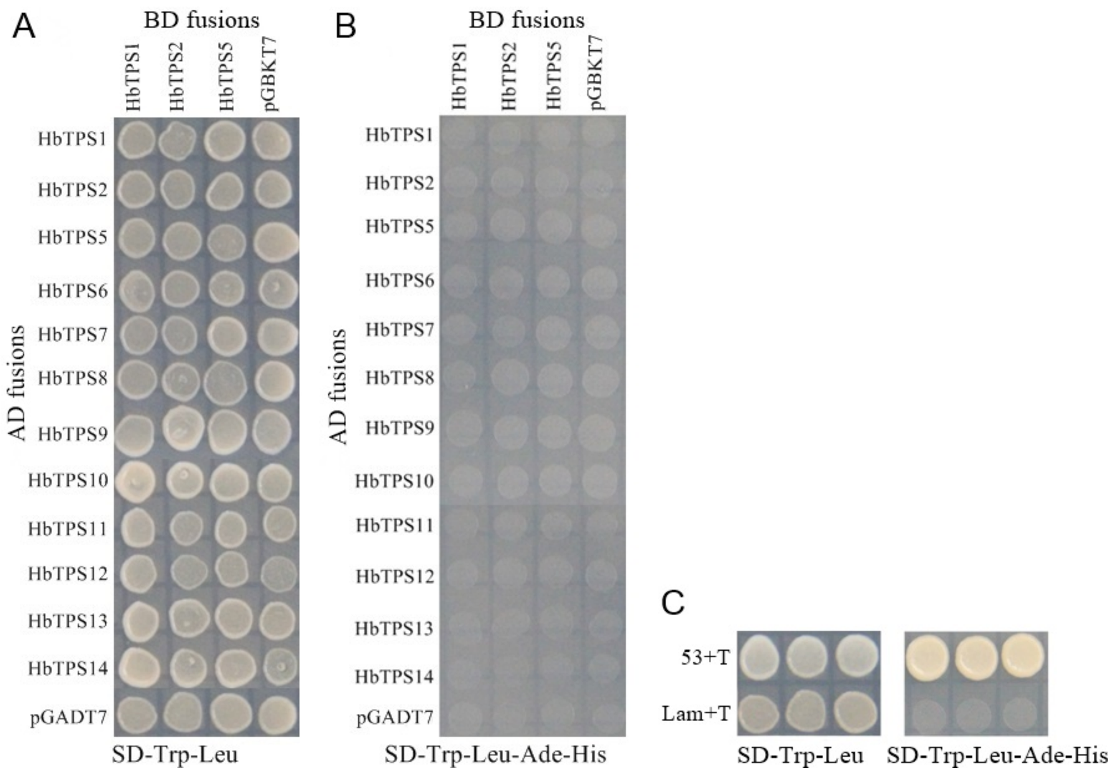

Fig. S4. Yeast two hybrid detection of pairwise interactions among HbTPS proteins. A, The growth status of all co-transformed Y2H-Gold strains on SD/-Trp/-Leu solid medium. B, The growth status of all co-transformed Y2H-Gold strains on SD/-Trp/-Leu/-His/-Ade solid medium. C, The growth status of Y2H-Gold strain containing positive control 53 plus T and negative control Lam plus T on SD/-Trp/-Leu and SD/-Trp/-Leu/-His/-Ade solid medium, respectively.

Tables:

Table S1 Basic information for the fourteen *HbTPS* genes and their predicted proteins

Gene name	GenBank accession no.	Deduced protein			
		CDS	AA	M.W. (kDa)	pI
HbTPS1	KT313591	2787	928	104.7	6.7
HbTPS2	KT313592	2823	940	106.0	6.5
HbTPS3	KT313593	2811	936	105.2	6.1
HbTPS4	KT313594	2793	930	104.9	6.7
HbTPS5	KT313595	2535	844	97.4	5.8
HbTPS6	KT313596	2586	861	97.5	6.0
HbTPS7	KT313597	2586	861	97.5	6.3
HbTPS8	KT313598	2538	845	95.2	5.4
HbTPS9	KT313599	2565	854	96.5	5.7
HbTPS10	KT313600	2565	854	96.4	6.1
HbTPS11	KT313601	2601	866	97.5	5.7
HbTPS12	KT313602	2565	854	96.6	5.7
HbTPS13	KT313603	2595	864	97.8	5.9
HbTPS14	KT313604	2529	842	95.6	6.5

Table S2 Nucleotide and amino acid identities among the coding sequences of 14 *HbTPS* cDNAs and their predicted proteins

aa / nt	*HbTPS1*	*2*	*3*	*4*	*5*	*6*	*7*	*8*	*9*	*10*	*11*	*12*	*13*	*14*
HbTPS1	—	93.3	73.4	74.2	28.6	27.4	26.6	28.9	28.7	26.9	26.9	28.2	26.7	26.4
2	92.6	—	73.1	74.0	27.3	27.3	26.8	28.9	29.2	27.2	27.0	28.4	27.1	26.4
3	72.6	72.7	—	88.5	27.8	27.5	26.8	29.4	30.5	27.0	27.1	30.3	27.0	26.6
4	72.6	72.8	90.6	—	27.8	27.0	26.5	28.8	29.2	27.4	26.4	28.8	26.6	26.4
5	41.3	41.4	43.6	43.1	—	63.5	63.3	70.4	67.6	75.3	95.8	66.7	63.2	60.5
6	43.0	43.2	42.5	44.1	64.8	—	76.5	65.9	66.3	59.1	63.4	65.6	92.5	72.5
7	42.7	41.8	42.9	43.6	63.2	75.4	—	66.6	65.6	60.7	63.1	64.7	76.9	87.5
8	42.6	42.6	42.6	42.2	68.0	66.0	65.6	—	76.7	65.0	68.6	76.6	65.4	63.6
9	41.6	42.5	42.8	42.6	66.3	63.9	64.4	71.6	—	63.0	66.4	95.7	65.8	61.8
10	42.2	41.8	41.6	42.2	72.9	63.4	63.6	65.6	64.8	—	74.9	62.4	59.0	57.7
11	43.0	42.5	43.7	43.2	94.5	65.0	63.6	66.4	65.5	72.7	—	65.4	62.7	60.4
12	41.5	41.4	42.3	42.7	66.2	64.4	64.2	72.3	93.8	65.5	65.5	—	65.1	61.3
13	42.4	42.8	42.5	43.3	64.1	92.7	75.3	65.8	64.2	62.9	64.4	64.2	—	72.7
14	36.7	35.8	33.6	34.3	61.1	72.9	89.9	64.0	60.7	60.7	60.8	60.6	71.3	—

Table S3 RNA-Seq analysis of the expression of *HbTPS1*, *2*, *5*, *11* and *12* in different Hevea tissues*

Tissue type	RPKM values				
	HbTPS1	*HbTPS2*	*HbTPS5*	*HbTPS11*	*HbTPS12*
Latex	53.63	37.25	167.55	25.32	38.21
Leaf	39.56	31.31	45.71	30.81	54.02
Seed	12.71	2.96	23.79	14.09	26.79
Female flower	48.94	18.69	35.40	30.11	81.38
Male flower	33.33	10.39	20.81	22.16	24.94
Bark	68.24	7.67	26.34	29.11	33.33
Root	12.89	7.71	18.62	14.15	69.54

* An average of 50 million Solexa ESTs for each Hevea tissue was employed in the analysis.

Table S5 Expression profile of nine SnKR1 marker genes in latex during tapping

Type in Arabidopsis	Gene name in Arabidopsis	Gene ID in Hevea	Gene expression (RPKM)			
			Tapping 1	Tapping 3	Tapping 5	Tapping 7
SnRK1-induced	ASN1	scaffold1123_27558	55.26	14.1	16.33	13.89
	bGAL	scaffold0398_821775	52.33	115.59	146.75	123.69
	AKINb	scaffold0111_1691081	15.16	7.04	5.1	6.53
	TPS8	scaffold1217_9194	4.73	1.65	1.85	1.41
	TPS10	scaffold0584_415760	69.26	8.88	9	5.97
SnRK1-repressed	UDPGDH	scaffold0137_967149	24.8	28.37	34.51	29.02
	MDH	scaffold1646_55411	18.81	86.82	100.33	102.06
	bZIP11	scaffold2020_7025	34.12	14.15	7.55	6.77
	TPS5	scaffold0282_458626	0.08	0.45	0.29	0.24

Table S6 Primer pairs used in amplifying full-length cDNAs of 12 *HbTPS* and 2 *HbSnRK1* genes, and partial genomic sequences of *HbTPS3* and *4*

Gene name	Sequence	Usage
HbTPS1	5'-TTTCTCTTGCCTTCTCTCTCTCTTT-3' (F)	Full-length cDNA amplification
	5'-CTGTGGTTTGATCCTGTTCTATTCT-3' (R)	
HbTPS2	5'-AGACTTCTTCTGGGCATTTGTTT-3' (F)	
	5'-TATGCTTTCCAAATCCAGACC-3' (R)	
HbTPS5	5'-GGGTTTGCATAGATCATTGTCTAT-3' (F)	
	5'-TTCCCCCTTTAAGACATAGCTCC-3'	
HbTPS6	5'-TCTCTGTCTATCCTTCCTCCCATT-3' (F)	
	5'-ACCCGTAAATGCGGGACTTA-3' (R)	
HbTPS7	5'-GCTAGTGAGGAGCCTCTTTCGT-3' (F)	
	5'-CGGTG GCATTGGTGTCAGG-3' (R)	
HbTPS8	5'-TGGGTAGACAAGAGGTTCCTTATTT-3' (F)	
	5'-GTGCAACAGAATTCGGACAAAC-3' (R)	
HbTPS9	5'-TCTTTCTTGTTTTAGAGCAACTTAGC-3' (F)	
	5'-GCTTCTCTAAAGTAACACTATGACGAT-3' (R)	
HbTPS10	5'-ATGATGTCAAGATCATGCACCAAT-3' (F)	
	5'-GGAAAAAGGAGGTTAAAATTCTATATA-3' (R)	
HbTPS11	5'-GGAGAGGGACCAAACCAAAC-3' (F)	
	5'-CCTCTTAGCTCGTGAGAACAAT-3' (R)	
HbTPS12	5'-TGCCCACTGTCAAGCAAAGA-3' (F)	
	5'-TCAACCTACATGCTTAAGGAGAA-3' (R)	
HbTPS13	5'-TTGTGGTGGGAAGCGTGC-3' (F)	
	5'-TTGTCCCAGTAAGTGGAATGTG-3' (R)	
HbTPS14	5'-AAACAAAAACTGGCAACAATGAT-3' (F)	
	5'-AAGAATACCAGTCAATCTCTCCCA-3' (R)	
HbSnRK1-1	5'-GGTTTTTGTCTCTTTGGCCTT-3' (F)	
	5'-GCTTTTACACAACAATCACCCATA-3' (R)	
HbSnRK1-2	5'-CGGAAAATGGATGGGTCAAC-3' (F)	
	5'-ATTAAAGCAGTACTCGAAAGTCAGA-3' (R)	
HbTPS3	5'-AACCACCCGCAGTTTTCAGT-3' (F)	Genomic DNA amplification
	5'-CTGCCAGCAAACCTTTCCTTA-3' (R)	
HbTPS4	5'-GCACTCCTGTCTCACCCAAAA-3' (F)	
	5'-ACCCTCAAGTCCAAGGATACG-3' (R)	

Table S7 Primer pairs used in qPCR analysis of *HbTPS* and *HbSnRK1* family genes, and *SnRK1* marker genes

Gene name	Sequence
HbTPS1	5'-TTTCTCTTGCCTTCTCTCTCTCTT-3'(F)
	5'-CAAAGGCACACAAGAATCAGC-3'(R)
HbTPS2	5'-TTATGCCTACCTTAACAGGAACC-3'(F)
	5'-TACAATTTGTTGATATACTCTAGGGC-3'(R)
HbTPS5	5'-AGGTTTTAGCGAGATACACTGG-3'(F)
	5'-TAAGACATAGCTCCTAGCATGAATAC-3'(R)
HbTPS6	5'-ATTGAAGTGGACCATTGGAGTAGA-3'(F)
	5'-ACCCGTAAATGCGGGACTTA-3'(R)
HbTPS7	5'-GCCACAGGGAGTTACTAAAGGTC-3'(F)
	5'-GGCGCTGAACAAAATGACAA-3'(R)
HbTPS8	5'-TTTGAATGTTTTGTGCTTGAGTGA-3'(F)
	5'-CAAAGACATAAAACCACCAAACG-3'(R)
HbTPS9	5'-TCTTTTCCCGCTTTTCTTTCAT-3'(F)
	5'-CAATAAGAAATAAAGAAAGAGGGGAA-3'(R)
HbTPS10	5'-TCTTTTCCCGCTTTTCTTTCAT-3'(F)
	5'-CAATAAGAAATAAAGAAAGAGGGGAA-3'(R)
HbTPS11	5'-GGAGAGGGACCAAACCAAAC-3'(F)
	5'-AGTTTGTATCTTTCCCTCTTTTGG-3'(R)
HbTPS12	5'-TCTTTTCCCGCTTTTCTTTCAT-3'(F)
	5'-CAATAAGAAATAAAGAAAGAGGGGAA-3'(R)
HbTPS13	5'-TCTTTTCCCGCTTTTCTTTCAT-3'(F)
	5'-CAATAAGAAATAAAGAAAGAGGGGAA-3'(R)
HbTPS14	5'-ATGGATACGGGCAAAAGAAGT-3'(F)
	5'-TCCCATGAAATAACTCAAAGGC-3'(R)
HbSnRK1-1	5'-TCCACAGACCTCTTCTTATGCC-3'(F)
	5'-GCTTTTACACAACAATCACCCA-3'(R)
HbSnRK1-2	5'-TCTATGGATCACGGACCTCTTGT-3'(F)
	5'-ATTAAAGCAGTACTCGAAAGTCAGAC-3'(R)
ASN1	5'-GCATGGGCCAAAAATCCTG-3' (F)
	5'-GCAGTCTTCTCTACAATCCCTTC-3'(R)

bGAL	5'-AGGCGTATTGCCATAGTTTTCT-3' (F)
	5'-AACCCAGGGATTTTCCAACT-3'(R)
AKINb	5'-GTAGCACTCAGCACCCTCCTT-3' (F)
	5'-AAACTACAACAGATCAAACGCAAG-3'(R)
TPS8	5'-ATTGAAGTGGACCATTGGAGTAGA-3' (F)
	5'-ACCCGTAAATGCGGGACTTA-3'(R)
TPS10	5'-AAGCGTGCTATCTTAATGAGTGA-3' (F)
	5'-GGCCCATAACCATCCAAATC-3'(R)
UDPGDH	5'-CTGCGGGTGAACTCGTTGA-3' (F)
	5'-GCAATGTAAGGTGTCGCAAAA-3'(R)
MDH	5'-TCTCTCTTGCTCTCCGTTATCG-3' (F)
	5'-GGAACTGCTTCAGAGCCGTAG-3'(R)
bZIP11	5'-AGGGATATGTAGAAGAACCAAGGA-3' (F)
	5'-ATAACAGCAGAAAACTACAACATCAT-3'(R)
YLS8	5'-CCTCGTCGTCATCCGATTC-3'(F)
	5'-CAGGCACCTCAGTGATGTC-3'(R)

Table S8 Primer pairs used in yeast complementation assay

Gene name	Sequence	Notes
HbTPS1、2	5'-CACCCGGGAAGCTTGTAAAAGAAATGCCTGGAAACCAGTACAACG-3' (F)<br>5'-CCGTCGACTCAAGAAGATGCATTGGCTAGTTT-3' (R)	Restriction enzyme cutting sites underlined; the added translational enhancement sequence shaded.
HbTPS5	5'-CACCCCCGGGAAGCTTGTAAAAGAAATGATGTCTAGATCGTATACCAATCTC-3' (F)<br>5'-CCGTCGACTCAATCTGAAGCATCTGCAAGA-3'(R)	
HbTPS6	5'-GCACCCGGGAAGCTTGTAAAAGAAATGGTGTCAAGATCGTGTGTGAAT-3' (F)<br>5'-GTGCGTCGACTCAAATAGCACTCTCAAAAGAAACC-3'	
HbTPS7	5'-GCACCCGGGAAGCTTGTAAAAGAAATGATGTCAAGATCTTGTATAAATTTG-3'<br>5'-GCGAGTCGACTCAGACGACATCATCGAAAGAAA-3' (R)	
HbTPS8	5'-GCACCCGGGAAGCTTGTAAAAGAAATGGTTTCAAGATCGTATTCAAAC-3' (F)<br>5'-GCGAGTCGACCTACTCTGAAGCATTGGCCAG-3' (R)	
HbTPS9	5'-GCACCCGGGAAGCTTGTAAAAGAAATGGTGTCGAGGTCATACTCAAA-3' (F)<br>5'-GCGAGTCGACTTACACGGTAACTGTTTGTTCTGA-3' (R)	
HbTPS10	5'-GCACCCGGGAAGCTTGTAAAAGAAATGATGTCAAGATCATGCACCA-3' (F)<br>5'-GTGAGTCGACCTATATATGGTAAAAAATTCTATGTAACTC-3' (R)	
HbTPS11	5'-GCAACTAGTAAGCTTGTAAAAGAAATGATGTCTAGATCGTATACCAATCT-3' (F)<br>5'-GTGACCCGGGTTAGGAAGCTGCAATTTCATTAAG-3'(R)	
HbTPS12	5'-GCACCCGGGAAGCTTGTAAAAGAAATGGTGTCAAGGTCATATTCTAATCT-3' (F)<br>5'-GTGAGTCGACTTACACTGTTACTGTTTGTTCTGAAAC-3'(R)	
HbTPS13	5'-GCACCCGGGAAGCTTGTAAAAGAAATGGTGTCTAGATCCTATATGAATTTT-3' (F)<br>5'-GTGAGTCGACTCAAATAATACTTTCGAAGGAGAC-3' (R)	
HbTPS14	5'-GCACCCGGGAAGCTTGTAAAAGAAATGTTGAATTTCCCTCGAAGTC-3' (F)<br>5'-GTGCGTCGACTTATTTGAAAGAAACTTGTACTTCTGC-3' (R)	
ScTPS1	5'-TCGACTAGTAAGCTTGTAAAAGAAATGACTACGGATAACGCTAAGG-3' (F)<br>5'-TCGCCCGGGTCAGTTTTTGGTGGCAGAGG-3' (R)	

Table S9 Primer pairs used in yeast two-hybrid analysis

Gene name	Sequence	Usage
HbTPS1, *2*	5'-CTGACCCGGGGATGCCTGGAAACCAGTACAACG-3' (F)	To establish pGBKT7 constructs
	5'-GTCAGTCGACTCAAGAAGATGCATTGGCTAGTTT-3' (R)	
HbTPS5	5'-CTGACCCGGGGATGATGTCTAGATCGTATACCAATCTC-3'(F)	
	5'-GTCAGTCGACTCAATCTGAAGCATCTGCAAGA-3' (R)	
HbTPS1, *2*	5'-CTGACCCGGGGATGCCTGGAAACCAGTACAACG-3' (F)	To establish pGADT7 constructs
	5'-GTCAGGATCCTCAAGAAGATGCATTGGCTAGTTT-3' (R)	
HbTPS5	5'-CTGACCCGGGGATGATGTCTAGATCGTATACCAATCTC-3'(F)	
	5'- GTCAGGATCCTCAATCTGAAGCATCTGCAAGA-3' (R)	
HbTPS6	5'-CTGACCCGGGGATGGTGTCAAGATCGTGTGTGAAT-3' (F)	
	5'-GTCACTCGAGTCAAATAGCACTCTCAAAAGAAACC-3' (R)	
HbTPS7	5'-CTGACCCGGGGATGATGTCAAGATCTTGTATAAATTTG-3'(F)	
	5'- GTCACTCGAGTCAGACGACATCATCGAAAGAAA-3' (R)	
HbTPS8	5'-CTGAGAATTCATGGTTTCAAGATCGTATTCAAAC-3' (F)	
	5'- GTCACCCGGGCTACTCTGAAGCATTGGCCAG-3' (R)	
HbTPS9	5'-CTGACCCGGGGATGGTGTCGAGGTCATACTCAAA-3'(F)	
	5'-GTCACTCGAGTTACACGGTAACTGTTTGTTCTGA-3'(R)	
HbTPS10	5'-CTGACCCGGGGATGATGTCAAGATCATGCACCA-3' (F)	
	5'- GTCACTCGAGTCAAGGCGAACTTCCTATTTCTT-3' (R)	
HbTPS11	5'-CTGACCCGGGGATGATGTCTAGATCGTATACCAATCT-3'(F)	
	5'-GTCACTCGAGTTAGGAAGCTGCAATTTCATTAAG-3'(R)	
HbTPS12	5'-CTGACCCGGGGATGGTGTCAAGGTCATATTCTAATCT-3'(F)	
	5'-GTCAGGATCCTTACACTGTTACTGTTTGTTCTGAAAC-3'(R)	
HbTPS13	5'-CTGACCCGGGGATGGTGTCTAGATCCTATATGAATTTT-3'(F)	
	5'-GTCACTCGAGTCAAATAATACTTTCGAAGGAGAC-3'(R)	
HbTPS14	5'-CTGACCCGGGGATGTTGAATTTCCCTCGAAGTC-3' (F)	
	5'-GTCACTCGAGTTATTTGAAAGAAACTTGTACTTCTGC-3'(R)	

Restriction enzyme cutting sites underlined.
